# Examination of the Prevalence of Female Athlete Triad Components among Competitive Cheerleaders

**DOI:** 10.3390/ijerph19031375

**Published:** 2022-01-26

**Authors:** Allison B. Smith, Jennifer L. Gay, Shawn M. Arent, Mark A. Sarzynski, Dawn M. Emerson, Toni M. Torres-McGehee

**Affiliations:** 1 School of Kinesiology, University of Louisiana at Lafayette, Lafayette, LA 70507, USA; 2 Department of Health Promotion & Behavior, University of Georgia, Athens, GA 30602, USA; jlgay@uga.edu; 3 Department of Exercise Science, University of South Carolina, Columbia, SC 29208, USA; sarent@mailbox.sc.edu (S.M.A.); sarz@mailbox.sc.edu (M.A.S.); torresmc@mailbox.sc.edu (T.M.T.-M.); 4 Department of Physical Therapy, Rehabilitation Science, and Athletic Training, University of Kansas Medical Center, Kansas City, KS 66160, USA; demerson@kumc.edu

**Keywords:** low energy availability, menstrual dysfunction, bone mineral density, female athlete triad

## Abstract

The purpose of this study was to examine individual and combined Female Athlete Triad components within collegiate cheerleaders, an at-risk group. Cheerleaders (*n* = 19; age: 20.3 ± 1.2 years) completed anthropometric measurements, health history questionnaires, resting metabolic rate, the eating disorder inventory-3 and symptom checklist, blood sample, and DXA scan. Participants completed dietary and exercise logs for 7 days and used heart rate monitors to track daily and exercise energy expenditure. Proportions were calculated for low energy availability (LEA) risk, disordered eating risk, and pathogenic behaviors. Chi-square analysis was used to determine the difference between cheerleaders who experience low EA with or without disordered eating risk. All cheerleaders demonstrated LEA for the days they participated in cheerleading practice, 52.6% demonstrated LEA with eating disorder risk and 47.4% demonstrated LEA without eating disorder risk, 52.6% self-reported menstrual dysfunction, 14% experienced menstrual dysfunction via hormonal assessment, and 0% demonstrated low bone mineral density. Overall, 47.7% presented with one Triad component, 52.6% demonstrated two Triad components using self-reported menstrual data, and 10.5% demonstrated two Triad components using hormonal assessments. All cheerleaders displayed LEA. These findings support the need for increased education on the individual components of the Triad and their potential consequences by qualified personal.

## 1. Introduction

Over the last two decades, competitive cheerleading has grown in popularity as a sport in both the adolescent and college aged populations, with estimates suggesting over a million annual participants [1,2]. This growth is a result of a transition from the previous role of cheerleaders who statically stood on the sidelines of sporting events promoting crowd involvement, to the now dynamic and engaging sport that places an emphasis on athletic and acrobatic skills. Due to this increase in full body, high intensity, athletic demands, competitive cheerleading is comparable to other sports such as gymnastics, ballet, swimming, and diving. These sports have a long-reported history of increased risk of eating disorders (EDs) due to the emphasis of lean body shape and the aesthetic nature of the activities required [3,4]. EDs also place those who participate in cheerleading and other aesthetic sports at risk for low energy availability (LEA), which is a component of the Female Athlete Triad (Triad) [5].

The Triad is defined by the American College of Sports Medicine as an interrelated condition that involves LEA with or without an ED, menstrual dysfunction, and low or abnormal bone mineral density (BMD) [5]. The Triad is associated with long-term health consequences, specifically complications that involve the cardiovascular, endocrine, reproductive, skeletal, gastrointestinal, renal, and central nervous systems, and mental health [5]. In 2014, the International Olympic Committee defined a new concept termed Relative Energy Deficiency in Sport (RED-S), which sought to expand the components of the Triad to include impaired metabolic rate, menstrual function, bone health, immunity, protein synthesis, and cardiovascular health [6]. The International Olympic Committee acknowledged that the three identified components of the Triad exist within the newly proposed syndrome and stressed the importance of examining the components individually. One main difference between the Triad and the RED-S descriptions is that within RED-S, LEA can be present when energy intake (EI) and total daily energy expenditure (TDEE) are balanced, which would indicate that there is not a deficit of energy present [6,7].

LEA, the precursor of other Triad components, is defined when a female reaches a level of energy availability (EA) ≤ 30 kcal/kg fat free mass (FFM) and can occur with or without a diagnosis of an ED [8]. While the exact etiology of LEA is not precisely known, EA may reach low levels by either unintentional or intentional methods [5,7,8,9,10]. Unintentionally achieved LEA is often due to the unawareness of proper sport specific needs or lack of education altogether on the physical and physiological needs for athletes [5,8,9]. Intentionally lowered EA can occur by increasing the overall exercise energy expenditure (EEE) to greater than EI or the inverse, lowering EI more than the energy expenditure. Methods for intentionally altering dietary intake can include pathogenic behaviors such as binge eating, purging, self-induced vomiting, use of diet pills or laxatives, engaging in excessive exercise, and fasting [5,11,12,13,14]. These pathogenic behaviors may morph into clinical EDs if not managed and treated appropriately.

EDs are mental health conditions classified by criteria in the Diagnostic and Statistical Manual of Mental Disorders fifth edition (DSM-5) and include anorexia nervosa, bulimia nervosa, binge eating disorder, other specified feeding and EDs, and unspecified feeding and EDs [14]. When an individual suffers from symptoms but fails to meet criteria outlined in the DSM-5, they are categorized as having disordered eating. These five categories were updated with the acceptance of the DSM-5 and allow for more precise inclusion and treatment of feeding and EDs. In addition to the reported complications of these disorders (irritability, insomnia [15], obsessive–compulsive behavior toward food [14], effects on the individual’s teeth, and electrolyte imbalance [16]), EDs have also been linked to psychological problems (depression, anxiety, and suicide). Additionally, prolonged energy restriction has been documented to compromise athletic performance in a variety of sports [5,7,8,11].

The second component of the Triad is menstrual dysfunction, which spans a continuum that ranges from healthy eumenorrhea to oligomenorrhea to functional hypothalamic amenorrhea [7]. Reproductive hormone function is most commonly recognized by amenorrhea, which is defined as the absence of menstrual cycles for more than three months [17]. The type of amenorrhea that is caused by LEA is termed functional hypothalamic amenorrhea, which is caused by a lack of the pituitary gland secreting luteinizing hormone (LH) at high frequencies [5,18]. There have been reports that when an individual suffers from LEA for as few as five days, there is a significant change in the LH pulsatility, which may lead to suppression of ovarian function and skeletal demineralization [18]. These complications have long-term consequences, such as infertility, which may be irreversible.

Abnormal or low BMD is the third component in the Triad, which ranges from optimal bone health to osteoporosis and is assessed through the gold standard measurement of a dual-energy X-ray absorptiometry (DXA) scan [5,7,19]. The International Society for Clinical Densitometry recommends that DXA scan results utilize the Z-score for diagnosis of osteoporosis, which compares an individual’s results to age and sex-matched controls. Osteoporosis, characterized by compromised bone strength and which predisposes individuals to fractures, is quantified by a Z-score of ≤−2.0. The precursor condition, osteopenia, is defined as a Z-score ranging from −1.0 to −2.0 [7]. Athletic participation generally positively impacts BMD, as shown by the findings of athletes reporting 5–15% higher Z-scores compared to nonathletes [5,20,21,22]. However, when athletes suffer from other components of the Triad, there are decreases in overall bone health and increased rates of diagnosed low BMD. Prevalence of low BMD in amenorrheic athletes ranges from 1.4% to 50.0% [23,24,25,26,27,28,29,30,31], which demonstrates the importance of early detection and treatment of all Triad components.

Triad research in sports has revealed a high prevalence of individual Triad components when the sport has an emphasis on leanness and aesthetic appeal [5]. Specifically, aesthetic sport athletes are 2–3 times more likely to suffer from the Triad [5]. There has been minimal research focused on the cheerleading population [2,32,33], with none being focused on cheerleading in the competitive area and with what has been presented being outdated and lacking a reflection of the new generation of the sport. This gap in the literature, coupled with the known lifelong health consequences that result from the Triad components and the large size of the cheerleading population, reinforces the need for updated research and recommendations for athletes, coaches, and governing organizations. Therefore, the purpose of this study was to examine the individual and combined Triad components of LEA with or without an ED risk, menstrual dysfunction, and low BMD within competitive college cheerleaders.

## 2. Materials and Methods

### 2.1. Participants

Twenty-one participants began the study, with 2 dropping out due to inability to complete all components of the study, yielding a total of 19 participants. College competitive cheerleaders (*n* = 19; age: 20.2 ± 1.24 years, height: 160.71 ± 7.7 cm; weight: 58.67 ± 7.75 kg) who were an active, participating, competitive member of a southeastern university cheerleading team participated in this study. Active and participating were defined as those cheerleaders who were members of the institution’s cheerleading team and who completed a minimum of three practices per week. The competitive aspect was defined as those who were training to be members of the University’s competitive team that would compete on the national level. This distinction adds additional practice time for the individual compared to other university cheerleaders who may only be required to attend games and social functions. Participants were excluded if they were unable to complete three practices per week during the time of the study collection, were unable to participate due to injury or illness, and were not a member of the college or institution. All 19 participants completed self-report measures related to menstrual dysfunction. However, only 14 participants were able and willing to provide a viable blood sample to be used in the hormonal analysis to determine menstrual function.

### 2.2. Instruments

#### 2.2.1. Demographic Survey

The basic demographic survey included age, education level, cheerleading participation history, current cheerleading experience, exercise background, and general medical history including menstrual cycle history. Additionally, self-reported measures related to weight included current weight, highest past weight, lowest past weight, ideal weight, and mental weight. Ideal weight is defined as the weight in which the individual would like to be at. Mental weight is defined as the perceived body weight in which the individual would reach if no conscious effort were made to control weight [34,35].

#### 2.2.2. Anthropometric Measurements

Multiple anthropometric measurements were collected including height (cm), weight (kg, self-report and measured), and body composition. Height was taken using a stadiometer (Shorr Productions, Olney, MD, USA) to the nearest 0.1 cm. Weight was measured wearing minimal clothes to the nearest 0.01 kg with a scale (Tanita, 331S, Tokyo, Japan). Body composition (FFM, fat mass, BMD) was assessed using Dual-Energy X-ray Absorptiometry (DXA; GE Lunar Prodigy densitometer).

#### 2.2.3. Dual-Energy X-ray Absorptiometry (DXA)

DXA was used to measure segmental BMD of the lumbar spine (L1–L4) total left hip, left femoral neck, and total body utilizing a GE Lunar Prodigy densitometer. Scoring of the BMD was taken from the established Z-score, which is the subject’s BMD score compared to the average scores of subjects who are the same age, sex, weight, and ethnic background. Z-scores that fall between −1.0 and +1.0 are considered normal BMD. A score below a −1.0 is described as a low BMD. A diagnosis of osteoporosis is applied when the z-score is below −2.0 [5,34].

#### 2.2.4. Resting Metabolic Rate (RMR)

RMR was measured using indirect calorimetry (Microlife MedGem; HealtheTech, Golden, CO, USA) to quantify energy expenditure by each participant at rest. The MedGem is a clinically validated measurement device that assesses RMR and has an interclass reliability range of 0.91–0.97 (mean = 0.94) [36,37]. While other methods to assess RMR are available, the MedGem is a quick, minimally invasive, and simple method.

#### 2.2.5. Eating Disorder Inventory-3 (EDI-3)

The EDI-3 is a self-reported measure used in identifying patients with ED risk patterns and consists of 91 items organized into 12 primary scales that include drive for thinness, bulimia, body dissatisfaction, low self-esteem, personal alienation, interpersonal insecurity, interpersonal alienation, interceptive deficits, emotional dysregulation, perfectionism, asceticism, and maturity fears. From these primary scales, the EDI-3 yields six composite scales, five general integrative psychological constructs (e.g., ineffectiveness, interpersonal problems, affective problems, over-control, and overall psychological maladjustment composite) and one ED specific composite (eating disorders risk composite). Test–retest reliability is high, with the ED risk coefficient at 0.98 and a median value of 0.95, and the general psychological maladjustment coefficient at 0.97, with a median value of 0.93; the current study reliability was 0.85.

Scoring of the EDI-3 utilizes a computer-based system that assesses outcomes for each participant. The scoring system provides an individualized report with raw scores, T scores, percentiles, and qualitative classifications, which include low clinical, typical clinical, and elevated clinical for all scales [38]. Ranges for classifications are based on percentile ranges for United States Adult Combined Clinical sample; low clinical ranges from the 1st to the 24th percentile, typical clinical ranges from the 25th to the 66th percentile, and elevated clinical ranges from the 67th to the 99th percentile [38]. To account for response style, such as irregular deviant or anomalous response patterns, to be considered at risk for ED using the EDI-3 participants had to be identified as having at least 2 or more composite scores in the “typical clinical” or “elevated clinical” classifications.

#### 2.2.6. Eating Disorder Inventory-3 Symptom Checklist (EDI-3 SC)

The EDI-3 SC is an additional self-report screening tool to help identify patients with ED risk patterns. This tool provides data regarding frequency of symptoms (i.e., binge eating; self-induced vomiting; exercise patterns; use of laxatives, diet pills, and diuretics) [39]. The tool range depends on whether participants report “yes” or “no” to engaging in behaviors and ranges between 10 to 42 questions. If a “yes” is denoted, additional follow-up questions related to frequency are answered. If a participant reports a “yes” to a specific behavior but then reports not engaging in the behavior in the last 3 months, this participant is not classified as at risk. To be considered at risk for ED on the EDI-3 SC, participants must meet criteria for at least one pathogenic behavior.

#### 2.2.7. Low Energy Availability (LEA) with or without an Eating Disorder (ED)

LEA was defined as EI–EEE relative to lean body mass in kilograms [8]. LEA was assessed on normal training days (three practices) over a seven consecutive day period. LEA was defined as <30 kcal/kg FFM. ED risk was defined if a participant reported an ED risk from the EDI-3 and/or the EDI-3 SC. LEA without an ED was defined as when a participant presented with LEA but was not deemed at risk for an ED from the EDI-3 and/or the EDI-SC.

#### 2.2.8. Energy Intake (EI)

EI was assessed from a 7-day diet log. Each participant was asked to document all food and beverages including description of the type of food, meal type (e.g., breakfast, lunch, dinner, snack), and the amount consumed for each of the 7 days. Participants logged their intake into the food processing software FoodProdigy (ESHA food processor 8.0, Salem, OR, USA), which provided breakdowns for total kilocalories (EI) and macronutrients consumption (e.g., carbohydrates, proteins, fats). Participants were provided this information if requested after the completion of the study.

#### 2.2.9. Exercise Energy Expenditure (EEE)

Three methods were used to determine EEE associated with cheerleading activities: (1) Polar Ignite watches, (2) Polar H10 heart rate chest monitors, and (3) for the exercise sessions where the participants were unable to wear the watch for the full duration of the exercise bout, researchers observed and documented activities using the compendium of physical activities by Ainsworth et al. [40] in order to determine the appropriate metabolic equivalent (MET). After the conversion to MET values, those values were used to calculate the energy expenditure by utilizing the Heyward [39] equation: EEE = duration (minutes) × ((METs × 3.5 × weight (kg))/200).

#### 2.2.10. Total Daily Energy Expenditure (TDEE)

Polar Ignite watches (Polar Electro Co., Woodbury, NY, USA) were worn continuously throughout the study to monitor TDEE and EEE. The watch is a non-invasive method of data capture that can be worn in all conditions (e.g., practice, lifting, swimming, showering). Various brands of heart rate monitors, including POLAR brand, accurately assess heart rate during rest and moderate activity (r > 0.90, SEE < 5 beats/min) [41,42].

#### 2.2.11. Self-Reported Menstrual Cycle Assessment

The demographic survey and EDI-3 SC were used to document age of menarche, frequency of cycles per year, frequency of missed cycles, history of disease, and use of birth control. Primary amenorrhea was defined as an absence of a menarche cycle by age 15 [5]. Secondary amenorrhea was defined as a cessation of a cycle after the original onset. These findings were determined based on answers from the EDI-3 questionnaires. To be classified at risk for overall self-reported menstrual dysfunction, participants had to be identified at risk for either primary amenorrhea alone, secondary amenorrhea alone, or a combination of both.

#### 2.2.12. Hormonal Menstrual Cycle Blood Assessment

Blood was collected following an overnight fast of at least 7 h. Participants were asked to sit comfortably while the sample was collected via venipuncture in the arm with a 21-gauge BD vacutainer needle and serum tube. All blood samples were allowed to clot at room temperature for 30 min followed by centrifuging for 15 min at 4 °C. Serum was aliquoted into 2-mL microtubes and stored at −80 °C until analysis. All serum samples were assayed for estradiol and analysis was completed by Bio Reference Laboratories (Elmwood Park, NJ). Bio Reference Laboratories utilize cut off points for the various menstrual cycle phases, which include follicular phase = 6.20–315.00 (pg/mL), ovulation phase = 28.60–525.00 (pg/mL), luteal phase = 7.69–752.00 (pg/mL), and postmenopausal ≤ 5.00–51.60 (pg/mL). For this study, participants were considered below the menstruating range if their serum sample was <5.00 (pg/mL).

### 2.3. Procedures

Approval for the study was obtained from the institutional review board, and all participants provided written consent prior to the study measures. After approval from the institutional review board, the research team members attended competitive cheerleading practices during February and March of 2021 on the campus of a Southeastern Conference university to share details and recruit participants for the study. It was during this time that the team was preparing for the national competition that would take place in April 2021. Cheerleaders who were interested in participating provided the research team with their contact information. During an informational meeting, participants provided consent then underwent laboratory testing to include anthropometric measurements, RMR, DXA scans, and all surveys. If the participant was fasted at that time, the blood sample was taken. If not fasted, blood was collected during the normal data collection week when a fasted sample could be obtained. Participants were provided written and verbal overviews of all study procedures, which included instructions on food and exercise logs and watch usage. Self-reported EI (foods and fluids) and planned and intentional exercise began the morning following the informational meeting and continued for seven consecutive days, which included three college competitive cheerleading practices. During the three practice days, EEE was calculated and used in conjunction with EI values to determine EA. Adherence to normal exercise and sport related activities (e.g., college cheerleading practices), as well as food and fluid intake were emphasized throughout the duration of the study. At the conclusion of the seven days, all research equipment was returned, and all logs were sent to the research team.

### 2.4. Statistical Analysis

Data were collected and input into SPSS statistical software (SPSS Inc., Version 27, Armonk, NY, USA) for all analyses. Alpha level was set at *p* < 0.05. Using G*Power Statistical software [43] for Chi-square analysis with a large effect size (0.7), the power calculation indicated a sample size of 17 participants was needed with estimated power being 0.80. Means and standard deviations were used for all demographic information and RMR, TDEE, EEE, and EA. Frequencies and proportions were calculated for EA risk, ED risk, and pathogenic behaviors. Chi-square analysis was used to determine the difference between cheerleaders who experience LEA with or without ED risk.

## 3. Results

### 3.1. Participant Characteristics

Twenty-one participants began the study, with two dropping out due to inability to complete all components of the study, yielding a total of 19 participants. Statistical power for chi square analysis was achieved when utilizing self-report data to categorize menstrual dysfunction. However, when using more reliable hormonal assessments for menstrual dysfunction, power was not achieved due to only 14 of the participants being able and willing to provide a blood sample. There were four instances where EEE values were not captured by the watches and the MET estimation was used to determine EA.

The distribution of ethnicity for all participants was 63.2% (*n* = 12) Caucasian, 15.8% (*n* = 3) African American, 10.5% (*n* = 2) Hispanic American, and 10.5% (*n* = 2) Multiethnic. Academic status was 10.5% (*n* = 2) freshman, 42.1% (*n* = 8) sophomore, 10.5% (*n* = 2) junior, and 36.8% (*n* = 7) senior. Cheerleaders identified their primary cheer position as flyer (31.6%, *n* = 6), base (47.4%, *n* = 9), and back spot (21.1%, *n* = 4). Additional physical measures are presented in Table 1.

### 3.2. Energy Needs Assessment and Low Energy Availability

The energy needs assessments (i.e., RMR, EI, EEE, EA) are presented in Table 2 and are as follows: RMR (1263.7 ± 147.6 kcals;), EI (1384.7 ± 391.8 kcals;), and EEE (746.0 ± 218.6 kcals;). The average EA for all 19 participants was 12.48 ± 8.01 kcals/kg FFM/day: with 100% (*n* = 19) being identified as being at a level of LEA.

### 3.3. Eating Disorder Risk and Pathogenic Behaviors

There were no cheerleaders who demonstrated ED risk by only the EDI-3; however, 42.1% (*n* = 8) were at risk according to the EDI-3 SC only, while 10.5% (*n* = 2) were at risk for ED according to both the EDI-3 and the EDI-3 SC. Further examination of the EDI-3 SC demonstrated 21.1% (*n* = 4) of cheerleaders reported engaging in 2 behaviors and 31.6% (*n* = 6) reported engaging in at least 1 behavior. Restricting was the most common behavior with 52.6% (*n* = 10) of cheerleaders self-identifying that they had engaged in restricting. Other behaviors that were reported included self-induced vomiting or purging (5.3%, *n* = 1), use of laxatives (10.5%, *n* = 2), and use of diet pills to control weight (5.3%, *n* = 1). Cheerleaders reported using planned and intentional exercise aside from cheerleading practice to maintain their weight, with 36.8% (*n* = 7) reporting they exercised to lose weight 25%-50% of the time. Pathogenic behaviors and percentage of time exercise used to control weight can be found in Table 3. Primary scales and composite scores for the EDI-3 are presented in Table 4.

### 3.4. Low Energy Availability with or without an Eating Disorder

Our study identified 52.6% (*n* = 10) of cheerleaders demonstrated LEA with an ED risk while 47.4% (*n* = 9) were at a level of LEA without an ED risk.

### 3.5. Menstrual Cycle Dysfunction

Overall, 52% (*n* = 10/19) of cheerleaders self-reported menstrual dysfunction in some capacity; 21.1% (*n* = 4/19) self-reported having experienced primary amenorrhea, and 52.6% (*n* = 10/19) self-reported secondary amenorrhea. Hormonal menstrual cycle assessment revealed 14.2% (*n* = 2/14) demonstrated below the 5.00 (pg/mL) threshold levels for estradiol indicating hormonal menstrual dysfunction. Cheerleaders reported 63.2% (*n* = 12/19) were currently taking a birth control method at the time of the study.

### 3.6. Bone Mineral Density

BMD values for total body, spine, legs, pelvis, segmental lumbar levels, total left femur, and left femoral neck are presented in Table 2. No participants were identified as having a z-score that would place them at risk for low BMD.

### 3.7. Triad Components

Overall, 47.7% (*n* = 9) of cheerleaders demonstrated one component of the Triad, while 52.6% (*n* = 10) demonstrated two components of the Triad when using self-reported menstrual data, and 10.5% (*n* = 2) demonstrated two components of the Triad when using the hormonal assessment data. No participants were identified as suffering from low BMD; therefore, the two components that were identified in our sample were LEA with or without an ED risk and menstrual dysfunction. Triad component frequencies and proportions are presented in Table 5.

## 4. Discussion

Competitive cheerleading is comparable in athletic demand to sports such as ballet, swimming, diving, figure skating, and gymnastics [3,4] Additionally, cheerleaders are at risk of suffering from components of the Triad due to the emphasis on physical appearance, lean body shape, low body weight, and also on elite athletic ability [1,2]. The purpose of this study was to examine individual and combined Triad components of LEA with or without an ED risk, menstrual dysfunction, and low BMD within competitive college cheerleaders. Our findings suggest competitive cheerleaders are at risk for Triad components, specifically LEA with or without ED and menstrual dysfunction.

### 4.1. Low Energy Availability

Overall, LEA was the most prevalent Triad component for cheerleaders, with 100% of the sample demonstrating below 30 kcal/kg FFM/day. LEA has been identified as being the most prevalent component of the Triad in other physically active populations and is the underlying cause for the physiological changes to both the reproductive system and bone, as well as declines in sport performance [5,6,7,34,44]. When compared to other athletic groups, our study had higher prevalence of LEA than gymnasts (44.8%), soccer players (33.3%), ballet dancers (22%) and volleyball players (20%) [45,46,47,48]. Similar results were found within synchronized swimmers, where 100% were also found to be at a level of LEA over the course of a 4-week study [49].

This is the first study to examine LEA within a sample of competitive cheerleaders, and the high rates of LEA within the sample may be a result of multiple factors. The Triad Coalition outlines four distinct pathways for LEA: disordered eating behaviors, a clinical ED, intentional weight loss without a clinical ED, or inadvertent undereating due to a lack of knowledge of caloric need for activity [7,50]. Athletes in general have 2–3 times higher risk for suffering an ED in sports that have weight classifications, aesthetic ideals, or require athletes to wear tight or revealing clothing. Those that deem a smaller body shape advantageous for the sport have been noted for even higher risk [26,32,51,52]. Competitive cheerleading is a highly aesthetic sport that is judged in a subjective manner and often has athletes wear revealing uniforms; therefore, it is reasonable that the cheerleaders may suffer from disordered eating or clinical EDs that could lead to LEA. LEA caused by intentional weight loss without an ED within this sample may be due to the period of data collection. This study was completed during the point in the season where the participants were preparing for the National competition for which they wanted to look their best. This factor may have led participants to engage in intentional weight loss but may not reflect their behaviors at other times in the season.

LEA caused by inadvertent undereating is a possible explanation within this sample. As stated previously, there has been no research to date to understand the energy needs for competitive cheerleading. Most cheerleaders and coaches may be uneducated on how to properly fuel for the sport’s demands. Further, our participants did not have access to registered dietitians or nutritionists who could educate team members on proper fueling, timing of meals, and weight management. Due to the high percentage of cheerleaders with LEA, having access to these resources would be highly beneficial. Athletes who are provided opportunities to increase their nutritional knowledge are more likely to consume high quality diets, including fruits, vegetables, and energy-providing carbohydrate-rich foods, which could help increase overall levels of EA [53,54,55].

### 4.2. LEA with or without an Eating Disorder Risk

More than half of our cheerleaders demonstrated LEA with an ED risk, whereas 47.2% revealed LEA without an ED risk. Overall prevalence of ED risk varies when examining athletes compared to non-athletic controls, with 18–20% of athletes and 5–9% non-athletes demonstrating ED behaviors [56,57]. This difference has become greater over the last decade with athletic population ED prevalence increasing approximately 10% [57,58,59,60,61]. When compared to other aesthetic sports, our findings are similar to results of literature focused on modern dancers (45%) [62] and equestrian athletes (42%) [52]. Band artists and cheerleaders are comparable due to both activities being involved in college athletics but often not receiving healthcare oversight or research focus [58]. The ED risk in these often-understudied populations was 70.7% for band artists [58] and 29.7% for auxiliary units (color guard, dance members, and majorettes) [63]. However, these studies utilized a different method for ED risk identification (i.e., the EDI-3 vs. the Eating Attitudes Test-26). When compared to previous cheerleading literature, 33% of cheerleaders were identified as being at risk for EDs [32], and our findings demonstrated a higher prevalence of ED risk. This increase from the 2012 study [32] may be explained by the increased athletic demands, media coverage, and overall athleticism of the sport.

In the current study, the EDI-3 and EDI-3 SC were used to assess ED risk along with other comorbid psychological factors. When using these tools in combination, the authors were able to examine traditional psychological constructs such as dieting, bulimia, and body image dissatisfaction, comorbid constructs such as self-esteem, maturity fears, and interpersonal alienation, while also examining pathogenic behaviors such as vomiting, use of diet pills, and over exercising [43]. Cheerleaders within the current study did not report the traditional ED risks; however, over 20% of the current participants reported in the “typical clinical” or “elevated clinical” category in four psychological scales, which included interpersonal insecurity, emotional dysregulation, perfectionism, and maturity fears, and two composite scales of interpersonal problems and over control.

The interpersonal insecurity scales assess discomfort and apprehension in silent situations, expressing personal feelings to others, the tendency to isolate, and a level of worry about rejection and hurt that is caused by a lack of support, trust, or protection [39]. For this specific sample of cheerleaders, the high proportion of risk from this scale may be attributed to multiple factors. The first may be due to the fact the cheerleaders are meant to engage with and encourage people to show school spirit in loud areas where there is little silence, which may lead these individuals to shy away from these quiet situations. A second explanation may be due to this study taking place during the COVID-19 pandemic, where periods of isolation were at an all-time high. The participants within this study all experienced a non-traditional academic year that led to a period where cheerleading practices were canceled prior to the study beginning. An additional factor may be attributed to a feeling of lack of support from the athletic administration stemming from the lack of cheerleading specific practice space and resources such as strength and conditioning coaches and registered dieticians.

The emotional dysregulation scale assesses mood instability, impulsivity, recklessness, anger, and self-destructiveness with the use of alcohol and drugs [35]. The high proportion of at-risk classifications could be explained from the sample, in which they were all current college cheerleaders. The maturation that occurs within the college years allows for individuals to reach the legal drinking age, which may increase the impulsive actions and increase the frequency of drinking. The perfectionism scale assesses the extent to which a person places a premium on achieving high goals with the highest possible standards for personal achievement [35]. Due to the subjective nature of the sport of cheerleading, where judges score teams based on how in sync each member is, there is a high level of perfectionism attributed to the sport. The goal within competitive cheerleading is to perform the routine perfectly with no judge’s deductions. These perfectionist traits are often seen as a motivator for cheerleaders and are used to enhance performance. However, these traits can quickly become unhealthy and have been documented in those who are at risk for EDs [64]. As cheerleaders strive to be perfect, there can be an increase in overall stress and anxiety, which may also exacerbate ED behaviors. Cheerleaders within this study may also have high perfectionism scores due to the feelings of always having to be perfect at practices. The participants within this study are all competing with over fifty other teammates for a limited number of spots at the end of year National competition.

The maturity fears scale assesses the desire to return to the security of childhood and is motivated by fears of psychosexual maturity [35]. Competitive cheerleading requires sport-specific training that begins at an early age. This specialized training allows for cheerleaders to be at an extremely elite skill level by the time they reach the college setting. Most of the participants within the current study were previously on competitive all-star or club cheerleading teams that were successful. Many participants were on teams who won the Cheerleading Worlds competition, which is thought to be the pinnacle event for the sport. With this success during their younger years, the high maturity fears may be explained by internal feeling of wanting to return to that previous time of cheerleading success. This desire to return to a young age may also impact wanting to be of a smaller body size, which can increase feelings of body image dissatisfaction and has been linked as a risk factor for ED in cheerleaders [33].

### 4.3. Pathogenic Behaviors

Our findings displayed competitive cheerleaders engaged in pathogenic behaviors, which included restricting, vomiting, or purging, use of laxatives, use of diet pills, and the overuse of exercise. These behaviors are commonly seen within college and elite athletes [51,60,65,66]. The current study findings demonstrate that cheerleaders present with higher rates than college athletes for dieting (52.6% vs. 15.6%) and laxative use (10.5% vs. 1.5%), similar rates for purging (5.3% vs. 2.9%), but lower rates for binge eating (0% vs. 18.6) [57]. When further examining uncommon populations within the literature, these pathogenic behaviors were also present among female body builders [67], band artists [58], and color guard members and majorettes [63]. In collegiate cheerleaders, lower rates were found for binge eating (0% vs. 11.8%), vomiting (5.3% vs. 9.6%), and use of laxatives (10.5% vs. 19.9%) when comparing the current study with previous cheer literature. Higher rates for use of exercise were found with 89.5% reporting using exercise to control weight ≥25% of time vs. 1.5% previously [32]. In the current study, cheerleaders did not have access to any formal strength and conditioning coaches or fitness instruction. This factor may indicate why the sample had high rates of utilization of exercise to control weight. With the increase in overall athletic ability in the sport of cheerleading, females may feel added pressure to stay fit to make college competitive cheer teams.

While none of our participants reported ED risk solely from the EDI-3, there were a large number that did indicate risk from either the EDI-3 SC alone or a combination of the EDI-3 and the EDI-3 SC. Some cheerleaders were at risk for LEA with an ED risk based off their risk for pathogenic behaviors. Pathogenic behaviors as well as the high classifications from psychological constructs (interpersonal insecurity, emotional dysregulation, perfectionism, and maturity fears) can lead to full ED behaviors over time. These findings present a unique opportunity to implement nutrition education, which can lead to a decrease in pathogenic behavior incidence [68]. Educational resources should be created for competitive college cheerleaders and coaches, which include fueling recommendations, information on ED traits (e.g., intense fear of gaining weight, distorted body image, and obsessive traits related to food intake), and resources available if there is a problem. Resources and information should be provided by a sports dietitian and/or health and safety personnel such as an athletic trainer or team physician/medical doctor [43].

### 4.4. Energy Needs Assessment

Overall EI in cheerleaders was 1384 ± 391.80 kcals, which is comparable to other college sports such as equestrian riders (1105 ± 164.2 kcals), softball players (1338.3 ± 313.5 kcals), and beach volleyball players (1281.2 ± 106.8 kcals), however this was lower than traditional volleyball (1785.6 ± 460.1 kcal) and women’s soccer (3214.3 ± 818.4 kcals) [43]. In comparison to aesthetic sports who are judged subjectively, cheerleaders’ EI was similar to college ballet dancers (1473.9 ± 312.5 kcals) [43], professional ballet dancers (1577 ± 89 kcals) [69], and gymnasts (1802 ± 289 kcals) [45].

When reviewing EEE, cheerleaders were measured to be at an average of 746 ± 218.64 kcals, which appears lower compared to other sports, specifically, soccer (1187.2 ± 39.7 kcals), volleyball (838.2 ± 77.6 kcals), and softball (811.2 ± 130.5 kcals) [43]. However, these other sports participate in practice 5–6 days per week where cheerleaders only practiced a total of 3 days (seven hours). If cheerleaders participated in additional practices, it is likely that the EEE would be higher than the previously reported averages for other sports. When considering RMR and EI, the values were similar, which demonstrates that they were consuming just enough energy to perform metabolic functions.

### 4.5. Menstrual Cycle Dysfunction

In the current study, more than half the participants were at risk for self-reported menstrual dysfunction, while only 14.2% were determined to exhibit hormonal menstrual dysfunction. Prevalence rates of menstrual dysfunction in athletic populations has been documented to range from 1–61% [70,71,72,73,74,75]. However, these studies only include clinical presentations or readily apparent menstrual dysfunctions. With the update to the Triad in 2007, there is a new focus on the subclinical conditions related to menstrual health that occur as an individual suffers from LEA [5]. Hormonal assay detection is the only accurate method to determine subclinical changes, due to dysfunction taking long periods of time to become notable by the individual. The literature has shown that within five [76] days of being at a level of EA ≤ 30 kcal/kg/FFM/day, there are subclinical changes that cause the pituitary gland to limit the amount of LH that is secreted [77]. Individuals who suffer from LH deficiency may be at further risk for infertility [5].

When examining the specific types of amenorrhea, our study identified 21.1% of cheerleaders self-reported primary amenorrhea, which is comparable to the 22% prevalence rate in a previous study including cheerleaders, gymnasts, and divers [78]. Secondary amenorrhea is widely more common and varies drastically with respect to sport type, age, training volume, and overall body weight of the individual [5]. Within the current study, 52.6% of cheerleaders presented with self-reported secondary amenorrhea, slightly lower than previous rates for dancers (69%) [79] and long distance runners (65%) [80] but higher than the general population (2–5%) [76,77,81].

With such a large majority of cheerleaders presenting with self-reported menstrual dysfunction and the long-term consequences associated with being amenorrheic, there is a need for education and intervention within this population. Educational efforts should be made to increase the awareness of the subclinical changes that occur within a female who is suffering from LEA and the potential life-long consequences. Recommendations should include proper fueling techniques to improve EA. Research has shown that an increase in overall EA from 20 to 30 kcal/kg/FFM/day restored amenorrheic athletes back to a normal menses [82].

### 4.6. Low Bone Mineral Density

Compared to other collegiate athletes, where 9.8% were classified as suffering from osteopenia and 1.8% were classified as osteoporotic [78], there were no cheerleaders who presented with low BMD. The BMD of an athlete is a direct reflection of an individual’s comprehensive history of EA and menstrual dysfunction coupled with their personal genetics and nutritional, behavioral, and environmental factors. These interrelated factors make it increasingly important to not only consider where the athlete is at currently within the BMD health continuum but also thinking about where they could be headed in the future [5]. Evidence suggests BMD decreases with each consecutive missed menstrual cycle and decreases may be irreversible [83,84,85,86]. Thus, there should be concern for future effects on BMD among our population considering the large percentage of our participants at risk for menstrual dysfunction.

There is a need for the collegiate cheerleading population, both athletes and coaches, to be informed about the importance of bone health with respect to the Triad and its components. Due to the increased athletic demands associated with the sport coupled with the potential BMD deficits due to the menstrual irregularities, there is a heightened risk for injuries, specifically stress fractures. There is a wealth of literature that indicates that stress fractures occur at a higher rate in physically active women with menstrual irregularities [87,88,89,90,91,92,93]. Additionally, there is a 2–4 times greater risk for amenorrheic athletes compared to eumenorrheic athletes of suffering a stress fracture during athletic participation [87].

### 4.7. Triad Components

There has been minimal research examining the prevalence of all three Triad components in any athletic or non-athletic population [25,26,78,94,95]. Of the few studies that did present all three components, the prevalence rates were low, ranging from 0–16% [25,26,94,95]. This is consistent with our findings of zero competitive cheerleaders presenting with all three Triad components.

### 4.8. Limitations and Future Research

Our study demonstrated a risk of Triad components, singularly as well as in combination, in competitive cheerleaders; however, some limitations should be appreciated. A large portion of the instruments used in this study are self-reported (EDI-3, EDI-3 SC, food and exercise logs, and menstrual history); therefore, we assume all participants responded accurately and truthfully. The instruments used to determine ED risk specifically serve only as an assessment of risk, rather than a clinical diagnostic tool. The gold standard for ED diagnosis includes an in-person interview by a mental health professional, which was not conducted in the current study. Second, we only used estimations and did not use the gold standard of doubly labeled water to assess exercise energy expenditure.

Additionally, our study included participants from only one college institution in the southeast United States. Competitive cheerleading spans the entire United States, as well as the world, and includes college-aged participants as well as adolescents. Therefore, the results of the current study cannot be generalized to the entire competitive cheerleading population. Future research should look to examine the Triad components within a larger sample that includes both college and adolescent ages and examine components within the male cheerleading population. It is also recommended that future research looks to compare prevalence and risks between college and adolescent cheerleaders.

A final limitation was the small sample size in which hormonal assessment for menstrual dysfunction was completed. The study was only able to collect blood samples from 14 participants, which did not meet power for the study and generalizations should be made with caution. Future studies should rely on blood assays rather than self-report measures for menstrual health due to the risk of oral contraceptives masking subclinical changes related to menstrual function.

## 5. Conclusions

This study provides the first look into the sport of competitive cheerleading and the Triad components. Overall, our study revealed that most competitive cheerleaders are at risk for LEA with a risk for ED and menstrual dysfunction but not for low BMD. These results provide important health-related information to athletes, coaches, school administrators, and competition organizers on the existence of the Triad components within this population. The rates we found between LEA with or without an ED or menstrual dysfunction support the need for increased education, oversight, and resources for cheerleaders. Appropriate evaluation of all three components should also be completed when an athlete is thought to be at risk or is diagnosed with any one component. Increased education and awareness to all three Triad components, LEA with or without and ED, menstrual dysfunction, and low BMD, needs to be implemented. The authors suggest that energy intake and proper fueling techniques should be the first focus due to the impact that overall EA has on the other components of the Triad.

## Figures and Tables

**Table 1:** Self-reported and measured physical measurements for cheerleaders (*n* = 19).

Anthropoemtric Measurement	All (*n* = 19)	
	Mean	SD
Height		
Measured Height (cm)	160.70	7.77
Weight		
Measured Weight (kg)	58.67	7.75
Highest Weight (kg)	62.12	8.50
Lowest Weight (kg)	54.67	6.67
Mental Weight (kg) *	60.95	11.15
Ideal Weight (kg)	56.38	6.26
Self-Reported Weight-Ideal Weight (kg)	−5.75	4.67
Mental Weight—Ideal Weight (kg)	4.57	7.14
Self-Reported—Weight-Mental Weight (kg)	39.02	22.11
Measured Weight-Mental Weight (kg)	−2.27	5.03
BMI		
Measured BMI (kg/m^2^)	22.62	1.96
Body Fat Percent		
DXA Body Fat (%)	26.11	4.19

* Mental Weight: Perceived weight if one did not consciously try to control weight.

**Table 2:** Energy need assessments, low energy availability risk, and bone mineral density scores for cheerleaders (*n* = 19).

Energy Needs Assessment	Mean	SD
Resting Metabolic Rate (kcals)	1263.68	147.58
Energy Intake (kcals)*	1384.7	391.80
Total Daily Energy Expenditure (kcals)	2467.60	426.95
Energy Balance (kcals)	−1043.14	580.59
Exercise Energy Expenditure (kcals) *	746.04	218.64
Energy Availability (kcals/kgFFM/day) *	12.48	8.01
Macronutrients		
Carbohydrates (g)	153.00	44.49
Carbohydrates (g/kg of body weight)	2.64	0.80
Proteins (g)	60.83	16.80
Proteins (g/kg of body weight	1.03	0.25
Fats (g)	57.82	19.81
Bone Mineral Density		
Total Z-score	1.7	0.74
Total Score (g/cm^2^)	1.2	0.11
Legs (g/cm^2^)	1.1	0.12
Spine (g/cm^2^)	1.1	0.13
Pelvis (g/cm^2^)	1.2	0.09
L1 vertebra (g/cm^2^)	1.3	0.15
L2 vertebra (g/cm^2^)	1.4	0.18
L3 vertebra (g/cm^2^)	1.4	0.15
L4 vertebra (g/cm^2^)	1.4	0.17
Left femoral neck (g/cm^2^)	1.1	0.11

Note: * Means and standard deviations are reflective of values calculated based on days where cheerleading practice was completed.

**Table 3:** Eating disorder characteristics among competitive cheerleaders (*n* = 19). Mean and standard deviation (SD) scores taken from the EDI-3 survey.

	Classification							
	Raw Score		Low Clinical		Typical Clinical		Elevated Clinical	
	Mean	SD	*N*	%	*N*	%	*N*	%
Eating Disorders Risk Scale								
Drive for Thinness	5.94	6.67	17	89.5	2	10.5	0	0
Bulimia	1.05	1.43	18	94.7	1	5.3	0	0
Body Dissatisfaction	10.36	8.22	17	89.5	2	10.5	0	0
Eating Disorder Risk Composite	97.47	17.75	19	100.0	0	0	0	0
Psychological Scale								
Low Self-Esteem	2.36	2.89	17	89.5	2	10.5	0	0
Personal Alienation	3.52	2.83	18	94.7	2	10.5	0	0
Interpersonal Insecurity	6.89	3.87	8	42.1	11	57.9	0	0
Interpersonal Alienation	4.78	4.32	15	78.9	3	15.8	1	5.3
Interceptive Deficits	5.21	6.81	17	89.5	1	5.3	1	5.3
Emotional Dysregulation	3.26	4.05	13	68.4	5	26.3	1	5.3
Perfectionism	12.52	5.91	8	42.1	6	31.6	5	26.3
Asceticism	3.42	3.22	17	89.5	2	10.5	0	0
Maturity Fears	8.31	4.33	7	36.8	8	42.1	4	21.1
Composite								
Ineffectiveness Composite	66.10	8.15	18	94.7	1	5.3	0	0
Interpersonal Problems Composite	82.21	12.40	14	73.7	4	21.1	1	5.3
Affective Problems Composite	76.89	15.25	17	89.5	1	5.3	1	5.3
Over control Composite	81.52	14.72	14	73.7	4	21.1	1	5.3
General Psychological Maladjustment	353.05	44.76	17	89.5	2	10.5	0	0

**Table 4:** Frequency (*n*) and percent (%) of pathogenic behaviors from the EDI-3 Symptom Checklist among cheerleaders (*n* = 19).

	All Data	
	*N*	%
Eating Behaviors	?	
Dieting	10	52.6
Binge Eating	0	0.0
Purging *	1	5.3
Laxatives	2	10.5
Diet Pills	1	5.3
Diuretics	0	0
Exercise to Control Weight		
0% of time	2	10.5
<25% of time	8	42.1
25–50% of time	7	36.8
More than 50% of time	1	5.3
100% of time	1	5.3

* Note Purging: self induced vomiting to control weight.

**Table 5:** Triad assessments with breakdown of spectrum components (energy availability, menstrual disturbance, and bone mineral density scores) for cheerleaders (*n* = 19).

Female Athlete Triad Risk	At Risk % (*N*)
1 component	47.4% (9)
2 components using self-report measures	52.6% (10)
2 components using hormonal assessment	10.5% (2)
Triad Risk Type	At Risk % (*N*)
LEA with ED Risk	52.6% (10)
Self-Reported Menstrual Dysfunction	52.6% (10)
Self-Reported Primary Menstrual Dysfunction	21.1% (4)
Self-Reported Secondary Menstrual Dysfunction	52.3% (10)
Hormonal Menstrual Dysfunction	14.2% (2)
Low Bone Mineral Density	0% (0)
LEA with Menstrual Dysfunction	52.6% (10)
EDI-SC Behavior Risk (e.g., binging, purging, dieting, laxatives, diet pills, etc.)	At Risk % (*N*)
Zero Behaviors	47.4 (9)
1 Behavior	31.6 (6)
2 Behaviors	21.1 (4)

Note: LEA for the week was calculated by taking the average of the 3 days of EA on practice days; (LEA) Low energy availability.
